# Infant with Intralobar and Extralobar Bilateral Pulmonary Sequestration Requiring Simultaneous Resection: A Case Report

**DOI:** 10.70352/scrj.cr.25-0282

**Published:** 2025-11-06

**Authors:** Kan Suzuki, Rina Matsuda, Masaaki Sato, Mariko Yoshida, Eiichiro Watanabe, Kotaro Tomonaga, Kazue Miyake, Jun Fujishiro

**Affiliations:** 1 Department of Pediatric Surgery, The University of Tokyo Hospital, Tokyo, Japan; 2 Division of Pediatric Surgery, Dokkyo Children’s Medical Center Tochigi, Shimotsuga, Tochigi, Japan; 3 Department of Thoracic Surgery, The University of Tokyo Hospital, Tokyo, Japan

**Keywords:** intralobar pulmonary sequestration, extralobar pulmonary sequestration, bilateral pulmonary sequestration, common drainage vein, simultaneous surgery

## Abstract

**INTRODUCTION:**

Coexisting bilateral intralobar and extralobar pulmonary sequestration has rarely been reported, with no previous reports indicating that simultaneous resection is necessary because of a common drainage vein.

**CASE PRESENTATION:**

A 1-year-old boy was prenatally suspected to have right pulmonary sequestration. A preoperative contrast-enhanced CT scan revealed coexisting right intralobar and left extralobar pulmonary sequestrations. The left extralobar sequestration drained into the right inferior pulmonary vein, which was shared by the right intralobar sequestration. The patient had a history of infection with right intralobar pulmonary sequestration during infancy, so a right lower lobectomy was considered. Although an isolated right lower lobectomy would have resulted in congestion of the left sequestrated lung, simultaneous bilateral thoracoscopic surgery was performed. First, thoracoscopic resection of the left sequestrated lung and ligation of the right feeding artery of the right sequestrated lung were performed with right differential lung ventilation, followed by thoracoscopic right lower lobectomy with left differential lung ventilation after repositioning the patient. The intra- and postoperative courses were uneventful.

**CONCLUSIONS:**

Simultaneous thoracoscopic surgery for bilateral pulmonary sequestration in infants with a common drainage vein is a reasonable strategy, as unilateral lesion resection may lead to postoperative complications.

## Abbreviations

BPS
: bilateral pulmonary sequestration
EPS
: extralobar pulmonary sequestration
IPS
: intralobar pulmonary sequestration
PS
: pulmonary sequestration
VATS
: video-assisted thoracic surgery

## INTRODUCTION

PS is a developmental lung bud abnormality in which an accessory pulmonary bud develops separately from the normal lung bud.^1)^ IPS occurs before the formation of the visceral pleura, whereas EPS occurs after the pleura has formed.^1)^ Coexistence of IPS and EPS is rare, and no previous reports have suggested the need for simultaneous resection due to the presence of a common drainage vein. Here, we report a case in which simultaneous resection of right IPS and left EPS was required after infection of the IPS.

## CASE PRESENTATION

**Fig. 1:**
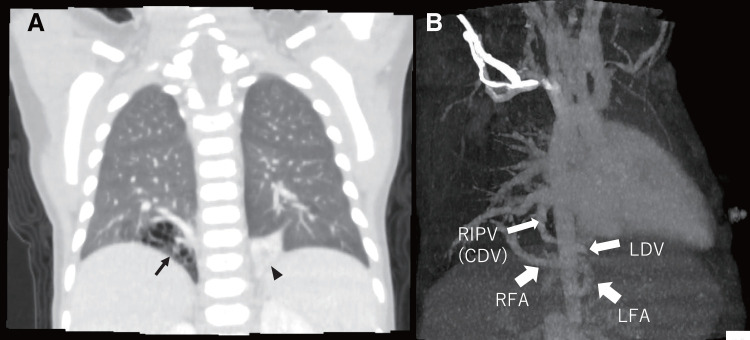
(**A**) Chest CT scan: Coronal section. The arrow indicates the right IPS, and the arrowhead indicates the left EPS. (**B**) 3D reconstruction image of CT scan: Feeding arteries and drainage veins in this patient. CDV, common drainage vein; EPS, extralobar pulmonary sequestration; IPS, intralobar pulmonary sequestration; LDV, left drainage vein; LFA, left feeding artery; RFA, right feeding artery; RIPV, right inferior pulmonary vein

**Fig. 2:**
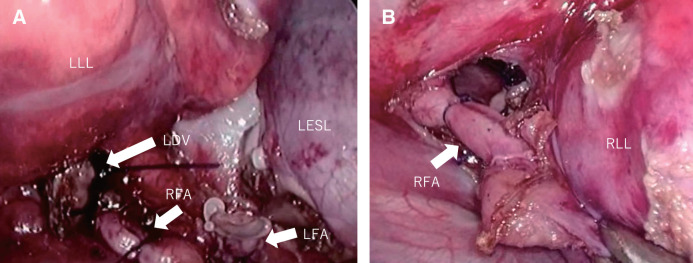
(**A**) Operative findings: Left thoracoscopic approach. The RFA was ligated after resection of the LESL. The vascular stumps of the LFA and LDV of the LESL are clearly visible. (**B**) Operative findings: Right thoracoscopic approach. The root of the RFA of the right IPS was ligated. IPS, intralobar pulmonary sequestration; LDV, left drainage vein; LESL, left extralobar sequestrated lung; LFA, left feeding artery; LLL, left lower lung; RFA, right feeding artery; RLL, right lower lung

A 1-year-old boy was prenatally suspected of having a right PS. Postnatal contrast-enhanced CT was performed to evaluate the lesion and confirmed the coexistence of a right IPS and a left EPS (**Fig. 1A**). The feeding arteries of the right IPS and the left EPS branched separately from the aorta, while the draining vein was a common inferior pulmonary vein (**Fig. 1B**). The patient experienced 2 episodes of infection in the right lower lobar IPS due to the accumulation of bronchial secretions or spread of inflammation in the normal right lower lobe, which required hospitalization and antibiotic treatment. Surgery was planned at 1 year of age. However, resection of only the right IPS using a right-sided surgical approach (right lower lobectomy) would require ligation of the left drainage vein, leading to congestion in the remaining left sequestrated lung. Therefore, we concluded that simultaneous resection of the right IPS and the left EPS was necessary. Because the left EPS was located entirely within the left thoracic cavity, it appeared to be challenging to remove the left EPS together with the right IPS using only a right-sided thoracoscopic approach. Thus, we decided to perform this operation using a bilateral thoracoscopic approach. First, left-sided sequestrated lung resection was performed with right differential lung ventilation. The left segmental lung was separated from the normal left lower lobe and mobilized. The proximal side of the feeding artery of the left EPS was ligated and then double-ligated with a Hem-o-lok; the peripheral side was ligated with a Hem-o-lok and then transected using a coagulator. The drainage vein was ligated with an endo-loop and resected just before the right mediastinal pleura to remove the left EPS. The feeding artery of the right IPS appeared in the field of view of the left thoracic approach and was ligated in advance (**Fig. 2A**). The patient’s position was then changed, and a right lower lobectomy was performed using a right thoracoscopic approach. The feeding artery of the right IPS was identified by the suture that had been previously ligated during the left thoracic approach and was then double-ligated with a Hem-o-lok and transected (**Fig. 2B**). Subsequently, a standard right lower lobectomy was performed. The right inferior pulmonary vein and the right lower lobe bronchus were transected using a linear stapler (ENDOPATH ETS-Flex 45; Ethicon, Johnson & Johnson, New Brunswick, NJ, USA). The operation lasted 688 min (including positional changes), and the intraoperative blood loss was 5 mL. Pathologically, the resected tissue on the right side showed IPS, with dilated bronchi in the sequestrated lung area and no bronchial communication with the normal lung area, while the resected tissue on the left side showed EPS, with the lung tissue being entirely collapsed but still retaining bronchi, arteries, and veins. The postoperative course was uneventful. The chest tubes were removed 2 days after surgery, and the patient was discharged on the 4th day. Two years after surgery, a CT scan showed no residual disease, the remaining lung had expanded well, and follow-up was completed.

## DISCUSSION

PS refers to the presence of abnormal lung tissue that differs from normal lung tissue and receives nutrition from the aorta. PS is divided into 2 types: IPS, in which the normal lung tissue is covered by the same pleura, and EPS, in which the normal lung tissue is covered by a separate pleura. In IPS, the draining veins are the pulmonary venous system, while in EPS, the draining veins are often the systemic venous system rather than the pulmonary venous system.^2)^ EPS is considered to have a congenital etiology and pathogenesis. However, the origin of IPS remains controversial. Some researchers suggest a congenital origin similar to that of EPS,^3,4)^ while others propose that intralobar lesions are acquired after various processes such as local infection, bronchial obstruction, and obstruction of the pulmonary circulation.^5,6)^ Some reports support the hypothesis of an acquired origin linked to the inhalation of foreign bodies or bacterial species.^7,8)^ In our patient, IPS and EPS coexisted and shared a common drainage vein. This supports the notion that both lesions may have originated from a common initial abnormality. Despite its benign nature, potential complications of PS are significant and include recurrent pulmonary infections, hemoptysis, congestive heart failure, and malignancy.^9)^ Surgery is indicated even in the neonatal period if the high blood flow in the feeding artery leads to heart failure in PS. In IPS, surgery is often indicated even if the patient is asymptomatic due to the risk of infection. On the other hand, in EPS, if the patient is asymptomatic or the lesions are shrinking, the patient may be observed. In our case, the right IPS had been infected twice during infancy, and resection of the right IPS was deemed necessary. Considering the growth of the remaining right and middle lobes, we planned a thoracoscopic right lower lobectomy at 1 year of age. Because the resection of the inferior pulmonary vein during right lower lobectomy simultaneously involves the resection of the drainage vein of the left EPS, there was concern about congestion in the left EPS. Therefore, simultaneous resection of right IPS and left EPS was necessary.

BPS is a rare congenital condition. Among the patients with BPS, 10 had coexisting IPS and EPS (**Table 1**).^2,3,10–16)^ Seven males and 3 females, including 7 children under 15 years of age and 3 adults, were reported. In 8 of these cases, IPS was located on the right side, while in 2 cases it was on the left; however, all EPS lesions were on the left side. Most patients with IPS received blood from the systemic circulation and drained into the pulmonary venous system; however, in 1 case,^14)^ the drainage vein was the azygous vein. In contrast, in most cases of EPS, vascular return is directed to the systemic venous circulation, and only those described by Spinella et al.^14)^ and our patient showed involvement of the pulmonary venous system. In the case reported by Spinella et al.,^14)^ the left pulmonary vein and the azygos vein were the draining veins of the EPS. Our patient differed from previous reports in that the drainage vein was common to both IPS and EPS. If the drainage vein of the left EPS was the right lower pulmonary vein, there are 2 possible explanations: an accessory pulmonary bud originating from the left pulmonary bud may have developed into an EPS, with its drainage vein connecting to the right lower pulmonary vein instead of a systemic vein; alternatively, an accessory pulmonary bud originating from the right pulmonary bud may have extended into the left thoracic cavity. In this patient, it is plausible that the left EPS shared a pleura with the right lower lobe and may have been part of a right IPS. During the 2nd episode of infection prior to surgery, the patient presented with an abscess in the right IPS and enlargement of the left EPS. At the time of surgery, strong adhesion was observed between the left EPS and the left lower lobe, suggesting that the infection had spread to the left EPS. These findings support the hypothesis that the left EPS originated from the right accessory lung bud.

**Table 1:** Reported cases of coexisting intralobar and extralobar pulmonary sequestration

Author	Sex	Age (years)	Site of IPS	FA and DV of IPS	Site of EPS	FA and DV of EPS	Operation method	Timing of operation
Pendse et al.^11)^	F	2.5	Right	FA; thoracic aorta DV; not detected	Left	FA; thoracic aorta DV; not detected	Thoracotomy	Metachronomous
Trudel et al.^12)^	M	15	Right	FA; thoracic aorta DV; azygos (same branch of EPS)	Left	FA; thoracic aorta DV; azygos (same branch of IPS)	Thoracotomy	Metachronomous
Kim et al.^13)^	M	10	Right	FA; thoracic aorta DV; right pulmonary vein	Left	FA; thoracic aorta DV; aberrant vein (the pulmonary or systemic nature of which could not be determined)	Thoracotomy	Metachronomous
Jeanfaivre et al.^3)^	M	22	Right	FA; aberrant from the aorta DV; pulmonary vein branch	Left	FA; from aberrant A of intralobar seq. DV; subphrenic vein	Posterolateral thoracotomy	Metachronomous
Spinella et al.^14)^	M	0	Right	FA; aberrant from the aorta DV; right pulmonary vein and azygos	Left	FA; aberrant from the aorta DV; left pulmonary vein and azygos	Thoracotomy	Metachronomous
Karakas et al.^15)^	F	0	Left	FA; aberrant from the celiac artery DV; inferior pulmonary vein	Left	FA; aberrant from the thoracic aorta DV; left brachiocephalic vein	Not mentioned	Sinchronomous
Yamamura et al.^16)^	M	41	Right	FA; aberrant from the aorta DV; inferior pulmonary vein	Left	FA; aberrant from aorta DV; not detected in operation	VATS	Sinchronomous
Schulz et al.^2)^	F	40	Left	FA; aberrant from the celiac axis DV; inferior pulmonary vein	Left (subdiaphragmatic)	FA; aberrant from the left gastric artery DV; not detected	VATS	Extralobar lesion consevertive
Parikh et al.^10)^	M	1	Right	FA; descending aorta DV; right pulmonary vein and portal vein	Left	FA; descending aorta DV; portal vein	VATS	Sinchronomous
Our Case	M	1	Right	FA; descending aorta DV; right pulmonary vein	Left	FA; descending aorta DV; right pulmonary vein	VATS	Sinchronomous

DV, drainage vein; EPS, extralobar pulmonary sequestration; F, female; FA, feeding artery; IPS, intralobar pulmonary sequestration; M, male; VATS, video-assisted thoracic surgery

In case of bilateral lesions, 5 cases reported in the 20th century underwent metachronous surgery,^3,11–14)^ while 4 patients underwent simultaneous surgery.^10,15–16)^ In 1 case,^2)^ only the IPS was resected, and the EPS was left under observation. The reduced surgical invasiveness of VATS has made simultaneous surgeries more feasible. However, in our patient, the drainage vein was completely shared; therefore, concerns were raised regarding the feasibility of metachronous surgery.

## CONCLUSIONS

In a patient with right IPS and left EPS sharing a common drainage vein as a congenital anomaly, simultaneous bilateral VATS was performed to prevent potential complications associated with the shared drainage vein.

## DECLARATIONS

### Availability of data and materials

All the related data are included with the article.
